# The virtual pivot point concept improves predictions of ground reaction forces

**DOI:** 10.3389/fbioe.2024.1286644

**Published:** 2024-03-26

**Authors:** Heiko Wagner, Oliver Schmitz, Kim J. Boström

**Affiliations:** Department of Movement Science, University of Münster, Münster, Germany

**Keywords:** computational movement science, ground reaction force, muscle-skeletal model, virtual pivot point, center of mass

## Abstract

Ground reaction forces (GRFs) are essential for the analysis of human movement. To measure GRFs, 3D force plates that are fixed to the floor are used with large measuring ranges, excellent accuracy and high sample frequency. For less dynamic movements, like walking or squatting, portable 3D force plates are used, while if just the vertical component of the GRFs is of interest, pressure plates or in-shoe pressure measurements are often preferred. In many cases, however, it is impossible to measure 3D GRFs, e.g., during athletic competitions, at work or everyday life. It is still challenging to predict the horizontal components of the GRFs from kinematics using biomechanical models. The virtual pivot point (VPP) concept states that measured GRFs during walking intercept in a point located above the center of mass, while during running, the GRFs cross each other at a point below the center of mass. In the present study, this concept is used to compare predicted GRFs from measured kinematics with measured 3D-GRFs, not only during walking but also during more static movements like squatting and inline lunge. To predict the GRFs a full-body biomechanical model was used while gradually changing the positions of the VPP. It is shown that an optimal VPP improves the prediction of GRFs not only for walking but also for inline lunge and squats.

## 1 Introduction

Ground reaction forces (GRFs) are crucial for the analysis of human movement. The measurement of GRFs during high-dynamic movements, such as sprinting, jumping, or cutting, is often conducted using 3D force plates fixed to the floor. These plates offer high accuracy, but they are mainly utilized in laboratory settings due to their design, cost, and limited functionality. Portable force plates, pressure plates and in-shoe pressure measurements, which measure only the vertical component of GRFs, can be used to analyze less dynamic movements, such as walking or squatting. Fong et al. (2008) was able to obtain satisfactory results for both vertical and anterior-posterior force components during walking while using pressure insoles. However, due to their design, functionality and price, force plates are mostly used in laboratory environments (Choi et al., 2013). In laboratories with a limited number of force plates, care must be taken by the subjects to hit the force plates properly with the foot, which therefore can influence the measurement (Challis, 2001). An outdoor measurement is difficult to implement due to the susceptibility of the systems to temperature and humidity (Psycharakis and Miller, 2006).

In scenarios where GRFs cannot be measured directly, such as athletic competitions, work, or everyday life, estimation of GRFs from kinematics using biomechanical models may be an option. However, accurate prediction of horizontal GRFs remains a challenge. In biomechanical laboratories, marker-based high-speed kinematic measurement systems are considered the gold standard, though camera-based marker-less or hybrid kinematic systems are catching up in terms of accuracy. However, both of these systems are limited by the calibrated area covered by the installed cameras.

Especially in large or cluttered spaces or outdoors, inertial measurement units (IMUs) can be used to measure kinematics. However, especially in these environments it is usually impossible to install force plates to measure the GRFs. It would be desirable, therefore, to predict the GRFs based on measured kinematics with IMUs. Studies with single IMUs (Neugebauer et al., 2012; Kiernan et al., 2018) showed that vertical ground reaction forces can be calculated, while the prediction of horizontal components is still a challenge. Under the assumption of a smooth transition between single and double support phases, the three-dimensional prediction of GRFs using multiple IMUs in full-body suits (Karatsidis et al., 2016) showed high correlations between measured and predicted GRFs.

In general, biomechanical models are susceptible to different sources of error, such as estimates of body segments (Pearsall and Costigan, 1999; Rao et al., 2006), joint parameters (Schwartz and Rozumalski, 2005), application and noise of the measurement system (Richards, 1999; Collins et al., 2009)), and estimates of the center of pressure (COP) (Schmiedmayer and Kastner, 1999). The estimation of GRFs from three-dimensional whole-body motion using the equations of motion further amplifies these inaccuracies. Audu et al. (2007) developed a model to estimate GRFs during stance, while Oh et al. (2013) used an artificial neural network to estimate GRFs and torques in gait analyses during single-support phases. The double support phases, on the other hand, involve splitting the GRFs between both feet and thus pose a major challenge for the simulation. Ren et al. (2008) calculated GRFs during double-support phases based on a smooth transition assumption.

Fluit et al. (2014) developed a universal model to predict GRFs and torques from three-dimensional whole-body movements in conjunction with a scaled musculo-skeletal model. For this purpose, the foot model was equipped with 12 virtual contact points to simulate the transition from non-contact to full contact between the ground and the foot, and to define the position of the center of pressure (COP). For walking, the correlations between the measured and predicted GRFs were very good to excellent, while for deep squat, stair ascend and stair descend the correlations for the antero-posterior and medio-lateral GRF components were rather low.

Skals et al. (2017) applied the method to sports-related movements, extending the number of artificial contact points from 12 to 18 and taking into account the thickness and the material composition of the shoe sole. For running, backwards running, side-cuts, vertical jumps and accelerations from standing position, very good correlations between measurement and estimation were achieved almost exclusively in the vertical and anterio-posterior GRF direction. For double-support phases or double-support phase transitions, the medio-lateral and transverse components of the GRF could only be estimated with rather low accuracy.

The basis to calculate the GRF are the Newton-Euler equations of motion, which state that the sum of all external forces acting at a body equals the acceleration of the center of mass (COM) times the body-mass *M*, i.e., the resulting force *F*_*COM*_ (Greenwood, 1988). This resulting force equals the GRF when no other external forces act on the body. Therefore, the calculated GRF has no lateral component during a squat because the lateral acceleration of the COM is close to zero. In contrast, the measured lateral forces at the left and right foot are not zero and in opposite directions.

These counteracting forces can be modeled by applying the concept of the virtual pivot point (VPP), which provides a fundamental framework for understanding human locomotion dynamics (Maus et al., 2010). During walking, the GRFs converge at a point above the center of mass, indicating a pivotal location, the VPP, where the forces intersect (Gruben and Boehm, 2012). The observed vertical position of the VPP varies between studies, due to the different walking speeds and curb height or manipulating the center of pressure (Müller et al., 2017; Vielemeyer et al., 2019; 2023a). In contrast, during running, the GRFs intersect below the center of mass, indicating a distinct biomechanical characteristic (Drama et al., 2020). To obtain a more general framework, Firouzi et al. (2019) extended the VPP concept by a subsystem of three inverted pendulums. In this study, we hypothesized that the VPP concept increases the accuracy of predicted GRFs, especially for the horizontal component. To test this hypothesis, we calculate the GRFs for different movements while varying the vertical position of the VPP.

## 2 Materials and methods

### 2.1 Experimental protocol

Eleven subjects (4 male and 7 female) with an average age of 24 ± 2.1 years and an average body height of 1.73 ± 0.11 cm were measured. The subjects were sports students at the University of Münster, physically healthy and without any significant previous musculo-skeletal diseases. All subjects were informed about the measurement process and gave their consent.

The movement tasks were chosen to cover the cases ‘double-support only’ during squats and inline lunges and ‘mixture of single and double support’ during walking. For inline lunge and squat the subjects started in front of the force plates, entered them after starting the measurement, while care was taken to ensure that each foot was on a separate force plate. Walking started 3 m in front of the force plates such that a uniform movement sequence could be established when stepping onto the force plates. During the movement process, care was taken to ensure that one of four force plates was loaded with each step. Practice tests were carried out before each measurement.

An inertial measurement unit (IMU) motion capture system (Xsens Technologies B. V., Enschede, Netherlands, sample frequency 120 Hz) with an MVN Link Suit and the software MVN Analyze^©^ was used to measure and analyze the kinematics. This IMU-system consists of 15 IMU-sensors, that are placed in a skin-tight Lycra suit at the head, sternum, pelvis, as well as the shoulders, upper arms, forearms, thighs, lower legs, and feet of both body sides. The kinematic IMU data were recorded and processed using proprietary algorithms of the XSens software (“HD” mode), yielding joint angle trajectories as recommended for biomechanical applications (Schepers et al., 2018) and validated in other studies (Krüger and Edelmann-Nusser, 2010). The data were exported to Matlab to be further analyzed.

The ground reaction forces (GRFs) were measured with an array of 8 force plates (Kistler Instrumente AG, Winterthur, Switzerland, sample frequency 1000 Hz, 60 × 90 cm). The GRFs consisted of the GRF vector for each foot touching the ground and its corresponding center of pressure (COP). The measurements were started simultaneously with a trigger-controlled synchronisation module. The data was exported to Matlab and filtered by a custom periodic median filter to remove humming noise. Then, the COP and force vector data were separately processed using a Butterworth filter with a cutoff frequency of 40 Hz and 20 Hz, respectively, to remove high-frequency noise.

### 2.2 Biomechanical model

**FIGURE 1:**
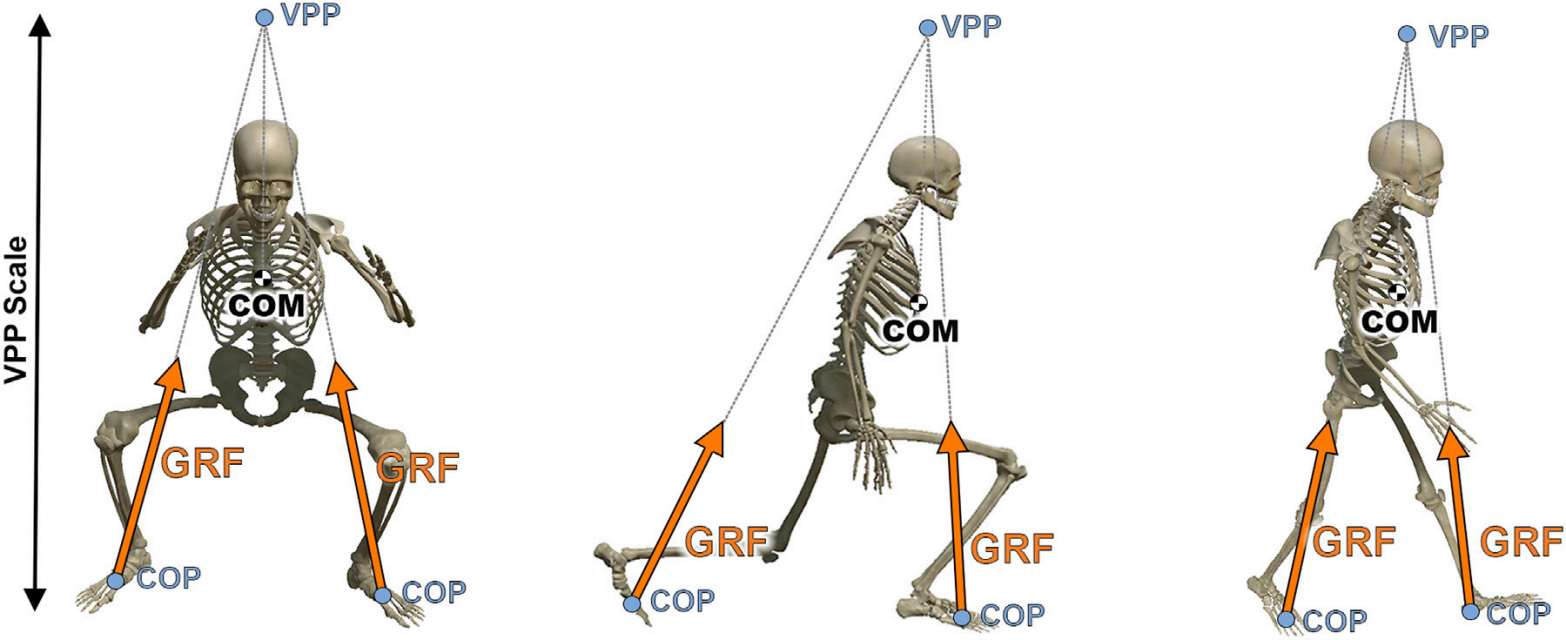
Visualisation of the model Myonardo^®^ (Predimo GmbH, Münster, Germany) during a squat, an inline lunge and during walking, from left to right respectively. Frontal and sagittal view of the positioning VPPscale of the virtual pivot point (VPP) in relation to the center of mass (COM) and the direction of the ground reaction forces (GRFs) starting from the center of pressure (COP) of each foot.

The biomechanical model Myonardo^®^ (version 4.3.3, Predimo GmbH, Münster, Germany) was used for the simulation (Wagner et al., 2022). Myonardo is developed in Matlab using the Simscape Multibody 3D simulation environment (Version 2021a, The MathWorks, Inc., Natick, Massachusetts, United States). It consists of 23 segments, 23 joints with 66 degrees of freedom, and 682 muscle-tendon units (Figure 1). The mass and inertia of each segment was scaled relative to the body mass and height (Hatze, 1980; Winter, 2009). The kinematic data was imported into the Myonardo^®^ from which the acceleration of the center of mass (COM) was determined and multiplied with the body-mass to calculate the resulting force vector *F*_COM_ acting at the COM.

### 2.3 Ground reaction forces calculation

In the following we will use a simplified notation. Mathematical objects may be denoted by more than one letter, occasionally subscripted by other letters or numbers, three-dimensional vectors will be in bold face, and their components will be denoted by the symbols *x*, *y* or *z* in brackets. For example, **COP**_*L*/*R*_ (*x*, *y*) is the 2-vector consisting of the *x* and *y* components of the 3-vector **COP**_*L*/*R*_, where the subscript *L*/*R* refers to the left and right foot, respectively. The Euclidean norm of a given vector ***v*** is denoted by $\|v\|=\sqrt{\sum_{k}v{\left(k\right)}^{2}}$ , where the sum is taken over the components of ***v***.

**FIGURE 2:**
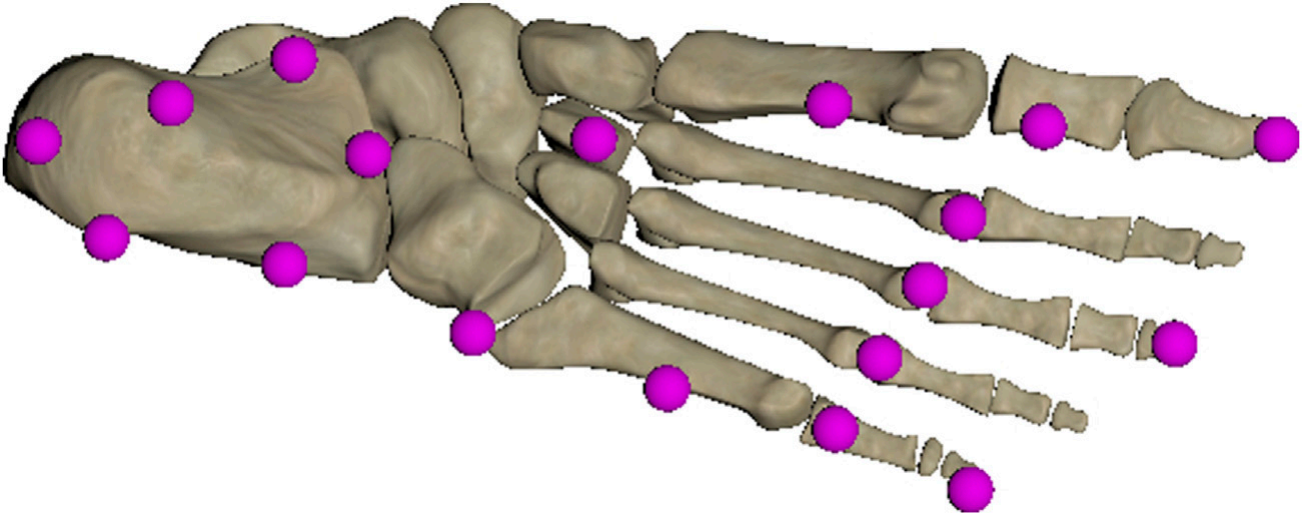
Schematic representation of the 18 contact points positioning to determine the contact between the foot and the ground surface of the Myonardo^®^ during a movement.

The calculation of the ground reaction forces (GRFs) for the left and right foot involved several steps. First, the center of pressure **COP**_*L*/*R*_ for the left and right foot was calculated (Eq. 1) following the method of Skals et al. (2017). To this aim, the foot model of the Myonardo^®^ was equipped with 18 contact points (Figure 2). The COPs of left and right foot were then calculated as

$${COP}_{L/R}=\frac{\sum_{i}W_{L/R,i}\;{CP}_{L/R,i}}{\sum_{j}W_{L/R,j}},$$ (1)

where **CP**_*L*/*R*,*i*_ and *W*_*L*/*R*,*i*_ are the position and relative weight, respectively, of contact point *i* of the left and right foot, respectively. The relative weight *W*_*L*/*R*,*i*_ of each contact point *i* was determined by,

$$W_{L/R,i}={WP}_{L/R,i}*{WV}_{L/R,i},$$ (2)

with the position and velocity transition functions calculated as, respectively^1^,

$${WP}_{L/R,i}=0.5\left(\cos\left(\pi *\frac{{CP}_{L/R,i}\left(z\right)/z_{\text{lim}}-0.8}{\left(1-0.8\right)}\right)+1\right)$$ (3)

$${WV}_{L/R,i}=0.5\left(\cos\left(\pi *\frac{\|{VCP}_{L/R,i}\left(x,y\right)\|/v_{\text{lim}}-0.15}{\left(1-0.15\right)}\right)+1\right),$$ (4)

where **CP**_*L*/*R*,*i*_(*z*) and ‖**VCP**_*L*/*R*,*i*_ (*x*, *y*)‖ are the vertical position and absolute horizontal velocity, respectively, of contact point *i* of the left and right foot, respectively, and with the position and velocity limits *z*_lim_ = 0.04 m and *v*_lim_ = 1.3 m/s, respectively. Eqs 2–4 create a smooth transition from zero to full ground contact for each contact point, with the parameters taken from Skals et al. (2017).

We used the inverse dynamic simulation of the Myonardo to obtain the force ***F***_COM_ applied to the whole body during movement which is equal to the body mass times the acceleration of the COM. The acceleration of the COM could also have been obtained directly from the IMU system, but since both methods provide only an estimate of the acceleration, and since these estimates may differ by an unknown amount as they are based on different models, we used the estimates solely from the Myonardo for consistency. Also, the Myonardo would enable the further addition of external forces or devices such as an exoskeleton, a basketball or a back pack.

During single-support phases the GRF is acting at the local COP of the foot with ground contact. During double-support phases we engage the virtual pivot point (VPP) method to calculate the GRFs acting at both feet as follows (Figure 1). The VPP is then assumed to be located above the COM with its (planar) xy-coordinates given by Eq. 5,

$$VPP\left(x,y\right)=COM\left(x,y\right)+\frac{COM\left(z\right)}{F_{\text{COM}}\left(z\right)}*F_{\text{COM}}\left(x,y\right),$$ (5)

and its (vertical) z-component given by Eq. 6,

$$VPP\left(z\right)=VPPscale*BH$$ (6)

with the body height BH and a scaling factor VPPscale that is to be optimized by varying VPPscale for the same movement and comparing it to the measured GRFs.

Next, for each foot we calculate the virtual pivot vector **VPV**_*L*/*R*_, which is the normalized vector pointing from the center of pressure to the virtual pivot point (Figure 1),

$${VPV}_{L/R}=\frac{VPP-{COP}_{L/R}}{\left\|VPP-{COP}_{L/R}\right\|}.$$ (7)

The ground reaction force vector of each foot (Eq. 8) is assumed to point in the direction of the virtual pivot vector, with its absolute value given by the yet unknown quantity GRF_*L*/*R*_, thus,

$${GRF}_{L/R}={GRF}_{L/R}*{VPV}_{L/R}.$$ (8)

Now, the COM force must equal the sum of both feet’s GRFs, so,

$$F_{\text{COM}}={GRF}_{R}+{GRF}_{L}.$$ (9)

Using (8) the above (Eq. 9) yields a set of linear equations that can be written in matrix form as Eq. 10,

$$F_{\text{COM}}=\left(\begin{array}{cc} {VPV}_{L} & {VPV}_{R} \end{array}\right)\left(\begin{array}{c} {GRF}_{L}\\ {GRF}_{R} \end{array}\right).$$ (10)

This set of linear equations can be solved with respect to the absolute GRF values of left and right foot, which can be written using the backslash operator ‘\’ as,

$$\left(\begin{array}{c} {GRF}_{L}\\ {GRF}_{R} \end{array}\right)=\left(\begin{array}{cc} {VPV}_{L} & {VPV}_{R} \end{array}\right)\backslash F_{\text{COM}}.$$ (11)

Inserting these solutions (Eq. 11) for GRF_*L*_ and GRF_*R*_ into (8) yields the estimated GRF vectors of left and right foot.

### 2.4 Data analysis

Walking is analysed from the first contact of the left foot with the force plate over the duration of a double step until the next contact of the left foot. Squat and inline lunge are considered from the instant when the COM is starting to move downward until it stops moving upwards. The VPPscale was varied between 0.2 and 2 in increments of 0.1. The root-mean-square error (RMSE) between the predicted and the measured GRF of each VPPscale in the vertical, medio-lateral and anterior-posterior directions were determined (Ren et al., 2008). Taking into account all directions and tasks, the optimal value for the VPPscale minimizing the RMSE was estimated visually.

## 3 Results

**FIGURE 3:**
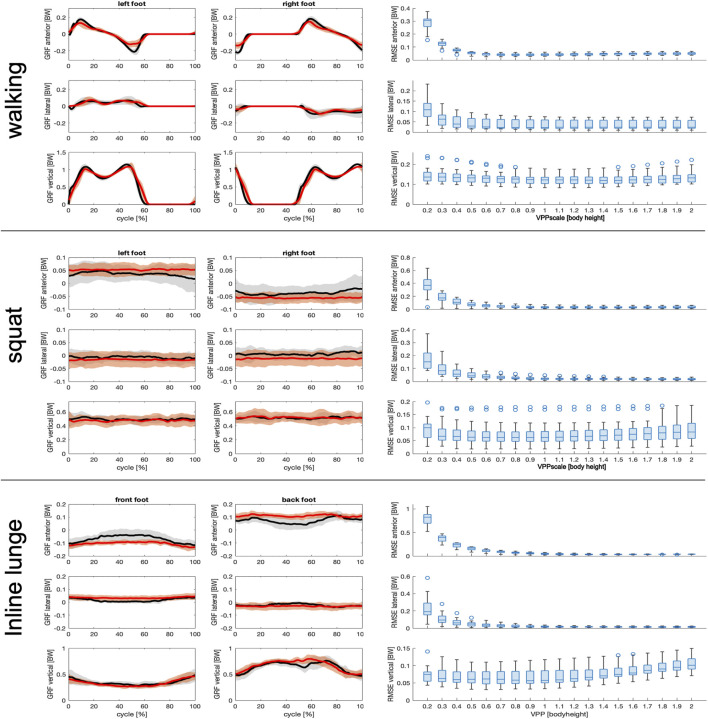
Left side: Comparison of directional components (anterior, lateral and vertical) of the measured ground reaction force (GRF) normalized to body weight (BW) (black with gray shading) with the corresponding predicted values (red with transparent shading) for VPPscale = 0.9 during walking (first row), squats (second row) and inline lunges (third row). The black lines show the mean values across all subjects and the shaded areas show the corresponding standard deviations. Right side: Box plots of the root mean square error (RMSE) of the predicted value relative to the measured values, while varying the position of the virtual pivot points (VPP) for all subjects, separately for the anterior, lateral and vertical direction. Within each box, the horizontal line denotes the median, and the box margins extend from the 0.25 to the 0.75 quantile (interquartile range, IQR). Vertical extending lines denote adjacent values within 1.5 IQR for all subjects, circles denote extreme values outside this range. Taking into account all directions and tasks, the optimal value for the VPPscale minimizing the RMSE was visually estimated as VPPScale = 0.9 (Table 1).

The measured and predicted GRFs of the walk cycles, the squats and the inline lunges are shown in Figure 3. As the variation of the VPPscale between 0.2 and 2 reaches a minimum RMSE at about 0.9 (Figure 3, right column), for all three movements a VPPscale = 0.9 is used to predict the GRFs (Figure 3, left column).

For all three movements, low VPPscale ≤0.8 largely affect the predicted GRFs in the antero-posterior and medio-lateral directions, compared to the measurement, whereas for VPPscale >0.8 the RMSE approaches nearly constant values (Figure 3; Table 1).

**TABLE 1:** Root-mean-square error of directional components of the ground reaction force (GRF) normalized to body weight (BW) as depicted in Figure 3. Displayed are the median and in brackets the interquartile range (IQR) of the 0.25 and 0.75 quartiles, of all subjects for VPPscale = 0.9.

RMSE (BW)	Walking	Squat	Lunge
antero-posterior	0.042 (0.034–0.047)	0.031 (0.021–0.046)	0.057 (0.044–0.071)
medio-lateral	0.023 (0.018–0.060)	0.023 (0.016–0.030)	0.020 (0.010–0.030)
vertical	0.124 (0.105–0.136)	0.062 (0.047–0.084)	0.057 (0.049–0.084)

The variation of the VPPscale only has a small influence on the predicted vertical GRFs, with lowest RMSE in the range 0.8 < VPPscale >1.4 (Figure 3; Table 1).

## 4 Discussion

The concept of the virtual pivot point (VPP) was originally based on findings during locomotion (Maus et al., 2010; Müller et al., 2010; Skals et al., 2017; Vielemeyer et al., 2019). The VPP is the result of complex control strategies depending on the movement goal and does not represent a single fixed point (Vielemeyer et al., 2019). In the present study we applied the VPP concept to improve GRF estimates especially for movements with vertical acceleration but lower horizontal dynamics, such as squatting and inline lunge. The GRF predictions of Fluit et al. (2014) are close to the measured GRFs for the case of walking, especially for the vertical and anterior-posterior components, and agree well with our predictions in terms of the RMSE. However, for squats, which is a movement task with reduced horizontal COM accelerations, our VPP-based approach is also able to predict the lateral forces and gives better RMSE values.

A limitation of the study is that it was not possible to compare the VPP estimated from the model simulations with the VPP calculated from the measured GRFs. For the experiment, we used the IMU system to measure the kinematics of the movements and the force plates to measure the GRF. The measurements were started simultaneously with a trigger-controlled synchronisation module, but there was no way to spatially align the two measurement systems, i.e., their world coordinate systems. During the calibration of the IMU system, we asked the subjects to stand in a position aligned with the anterior-posterior direction of the force plates. This allowed us to compare the three spatial directions of the measured and predicted GRF, while the absolute position of both systems relative to each other remained unknown. Therefore, it was not possible to relate the measured GRFs provided by the force plate system to the COM-based coordinate system provided by the IMU system, so a VPP based on the measured GRFs could not be calculated.

We found that for all measured tasks the RMSE becomes much less sensitive for VPPscale >0.8, so the exact position of the VPP does not seem to have much effect on the calculation of the GRF above this threshold. This may be explained by the angle between the connection lines of the VPP with the COPs of the two feet (***VPV***_L∕R_ in Eq. 7) becomes less sensitive on the VPP height. Furthermore, we found that during walking, the RMSE in the lateral direction does not change very much for values above VPPscale >0.5. This is compatible to the finding of Firouzi et al. (2019), who calculated the VPP in the frontal plane to be slightly below the COM. While we base our analysis on the assumption that the VPP is similar for the single and double support phases of walking, Vielemeyer et al. (2021) found that the VPP is significantly above the COM during the single support phase, whereas the VPP is below the COM during the double support phase. Therefore, when calculating the GRFs for walking, different positions of the VPP during double and single support should be considered in a future study. Furthermore, while walking with bent legs which can be compared with the lunge posture of the present study, Vielemeyer et al. (2023b) observed a (cranial) shift in the VPP position.

In conclusion, especially for movements with less horizontal accelerations, e.g., squat and inline lunge, the concept of a VPP improves the prediction of the three-dimensional GRFs.

## Data Availability

The raw data supporting the conclusion of this article will be made available by the authors, without undue reservation.

## Footnotes

1 We corrected an obvious typo in the original paper by Skals et al. (2017), where *π* was accidentally placed in the denominator instead of the numerator.

## References

[B1] Audu M. L. Kirsch R. F. Triolo R. J. (2007). Experimental verification of a computational technique for determining ground reactions in human bipedal stance. J. Biomechanics 40, 1115–1124. 10.1016/j.jbiomech.2006.04.016 16797023

[B2] Challis J. H. (2001). The variability in running gait caused by force plate targeting. J. Appl. Biomechanics 17, 77–83. 10.1123/jab.17.1.77

[B3] Choi A. Lee J.-M. Mun J. H. (2013). Ground reaction forces predicted by using artificial neural network during asymmetric movements. Int. J. Precis. Eng. Manuf. 14, 475–483. 10.1007/s12541-013-0064-4

[B4] Collins S. H. Adamczyk P. G. Ferris D. P. Kuo A. D. (2009). A simple method for calibrating force plates and force treadmills using an instrumented pole. Gait Posture 29, 59–64. 10.1016/j.gaitpost.2008.06.010 18755590 PMC2665306

[B5] Drama z. Vielemeyer J. Badri-Spröwitz A. Müller R. (2020). Postural stability in human running with step-down perturbations: an experimental and numerical study. R. Soc. Open Sci. 7, 200570. 10.1098/rsos.200570 33391782 PMC7735328

[B6] Firouzi V. Seyfarth A. Ahmad Sharbafi M. (2019). Does vpp exist in lateral balancing? In *9 International Symposium on Adaptive Motion of Animals and Machines (AMAM 2019)* . CONF.

[B7] Fluit R. Andersen M. S. Kolk S. Verdonschot N. Koopman H. F. J. M. (2014). Prediction of ground reaction forces and moments during various activities of daily living. J. Biomech. 47, 2321–2329. 10.1016/j.jbiomech.2014.04.030 24835471

[B8] Fong D. T.-P. Chan Y.-Y. Hong Y. Yung P. S.-H. Fung K.-Y. Chan K.-M. (2008). Estimating the complete ground reaction forces with pressure insoles in walking. J. Biomechanics 41, 2597–2601. 10.1016/j.jbiomech.2008.05.007 18571656

[B9] Greenwood D. (1988). Dynamics of a rigid body. Princ. Dyn., 389–468.

[B10] Gruben K. G. Boehm W. L. (2012). Mechanical interaction of center of pressure and force direction in the upright human. J. Biomech. 45, 1661–1665. 10.1016/j.jbiomech.2012.03.018 22521240

[B11] Hatze H. (1980). A mathematical model for the computational determination of parameter values of anthropomorphic segments. J. Biomechanics 13, 833–843. 10.1016/0021-9290(80)90171-2 7462257

[B12] Karatsidis A. Bellusci G. Schepers H. M. de Zee M. Andersen M. S. Veltink P. H. (2016). Estimation of ground reaction forces and moments during gait using only inertial motion capture. Sensors Basel, Switz. 17, 75. 10.3390/s17010075 PMC5298648 28042857

[B13] Kiernan D. Hawkins D. A. Manoukian M. A. C. McKallip M. Oelsner L. Caskey C. F. (2018). Accelerometer-based prediction of running injury in national collegiate athletic association track athletes. J. Biomech. 73, 201–209. 10.1016/j.jbiomech.2018.04.001 29699823 PMC6561647

[B14] Krüger A. Edelmann-Nusser J. (2010). Application of a full body inertial measurement system in alpine skiing: a comparison with an optical video based system. J. Appl. Biomechanics 26, 516–521. 10.1123/jab.26.4.516 21245513

[B15] Maus H.-M. Lipfert S. W. Gross M. Rummel J. Seyfarth A. (2010). Upright human gait did not provide a major mechanical challenge for our ancestors. Nat. Commun. 1, 70. 10.1038/ncomms1073 20842191

[B16] Müller R. Grimmer S. Blickhan R. (2010). Running on uneven ground: leg adjustments by muscle pre-activation control. Hum. Mov. Sci. 29, 299–310. 10.1016/j.humov.2010.01.003 20304516

[B17] Müller R. Rode C. Aminiaghdam S. Vielemeyer J. Blickhan R. (2017). Force direction patterns promote whole body stability even in hip-flexed walking, but not upper body stability in human upright walking. Proc. Math. Phys. Eng. Sci. 473, 20170404. 10.1098/rspa.2017.0404 29225495 PMC5719626

[B18] Neugebauer J. M. Hawkins D. A. Beckett L. (2012). Estimating youth locomotion ground reaction forces using an accelerometer-based activity monitor. PloS one 7, e48182. 10.1371/journal.pone.0048182 23133564 PMC3485031

[B19] Oh S. E. Choi A. Mun J. H. (2013). Prediction of ground reaction forces during gait based on kinematics and a neural network model. J. Biomech. 46, 2372–2380. 10.1016/j.jbiomech.2013.07.036 23962528

[B20] Pearsall D. Costigan P. (1999). The effect of segment parameter error on gait analysis results. Gait Posture 9, 173–183. 10.1016/S0966-6362(99)00011-9 10575078

[B21] Psycharakis S. G. Miller S. (2006). Estimation of errors in force platform data. Res. Q. Exerc. Sport 77, 514–518. 10.1080/02701367.2006.10599386 17243226

[B22] Rao G. Amarantini D. Berton E. Favier D. (2006). Influence of body segments’ parameters estimation models on inverse dynamics solutions during gait. J. Biomechanics 39, 1531–1536. 10.1016/j.jbiomech.2005.04.014 15970198

[B23] Ren L. Jones R. K. Howard D. (2008). Whole body inverse dynamics over a complete gait cycle based only on measured kinematics. J. Biomechanics 41, 2750–2759. 10.1016/j.jbiomech.2008.06.001 18672243

[B24] Richards J. G. (1999). The measurement of human motion: a comparison of commercially available systems. Hum. Mov. Sci. 18, 589–602. 10.1016/S0167-9457(99)00023-8

[B25] Schepers M. Giuberti M. Bellusci G. (2018). enXsens MVN: consistent tracking of human motion using inertial sensing. Tech. Rep. 10.13140/RG.2.2.22099.07205

[B26] Schmiedmayer H.-B. Kastner J. (1999). Parameters influencing the accuracy of the point of force application determined with piezoelectric force plates. J. Biomechanics 32, 1237–1242. 10.1016/S0021-9290(99)00109-8 10541075

[B27] Schwartz M. H. Rozumalski A. (2005). A new method for estimating joint parameters from motion data. J. Biomechanics 38, 107–116. 10.1016/j.jbiomech.2004.03.009 15519345

[B28] Skals S. Jung M. K. Damsgaard M. Andersen M. S. (2017). Prediction of ground reaction forces and moments during sports-related movements. Multibody Syst. Dyn. 39, 175–195. 10.1007/s11044-016-9537-4

[B29] Vielemeyer J. Grießbach E. Müller R. (2019). Ground reaction forces intersect above the center of mass even when walking down visible and camouflaged curbs. J. Exp. Biol. 222, jeb204305. 10.1242/jeb.204305 31266780

[B30] Vielemeyer J. Müller R. Staufenberg N.-S. Renjewski D. Abel R. (2021). Ground reaction forces intersect above the center of mass in single support, but not in double support of human walking. J. Biomechanics 120, 110387. 10.1016/j.jbiomech.2021.110387 33798969

[B31] Vielemeyer J. Schreff L. Hochstein S. Müller R. (2023a). Virtual pivot point: always experimentally observed in human walking? PLOS ONE 18, e0292874. 10.1371/journal.pone.0292874 37831656 PMC10575527

[B32] Vielemeyer J. Staufenberg N.-S. Schreff L. Rixen D. Müller R. (2023b). Walking like a robot: do the ground reaction forces still intersect near one point when humans imitate a humanoid robot? R. Soc. Open Sci. 10, 221473. 10.1098/rsos.221473 37266041 PMC10230186

[B33] Wagner H. Boström K. J. de Lussanet M. de Graaf M. L. Puta C. Mochizuki L. (2022). Optimization reduces knee-joint forces during walking and squatting: validating the inverse dynamics approach for full body movements on instrumented knee prostheses. Mot. Control 27, 161–178. 10.1123/mc.2021-0110 36252948

[B34] Winter D. A. (2009). Biomechanics and motor control of human movement. 4th ed. edn. Hoboken, NJ, USA: John Wiley and Sons, Inc. 10.1002/9780470549148

